# Axial Displacements and Removal Torque Changes of Five Different Implant-Abutment Connections under Static Vertical Loading

**DOI:** 10.3390/ma13030699

**Published:** 2020-02-04

**Authors:** Ki-Seong Kim, Young-Jun Lim

**Affiliations:** Department of Prosthodontics and Dental Research Institute, School of Dentistry, Seoul National University, Seoul 03080, Korea; namsang0249@nate.com

**Keywords:** dental implants, implant-abutment connection, settling effect, static loading, removal torque

## Abstract

The aim of this study was to examine the settling of abutments into implants and the removal torque value under static loading. Five different implant-abutment connections were selected (Ext: external butt joint + two-piece abutment; Int-H2: internal hexagon + two-piece abutment; Int-H1: internal hexagon + one-piece abutment; Int-O2: internal octagon + two-piece abutment; Int-O1: internal octagon + one-piece abutment). Ten implant-abutment assemblies were loaded vertically downward with a 700 N load cell at a displacement rate of 1 mm/min in a universal testing machine. The settling of the abutment was obtained from the change in the total length of the entire implant-abutment unit before and after loading using an electronic digital micrometer. The post-loading removal torque value was compared to the initial torque value with a digital torque gauge. The settling values and removal torque values after 700 N static loading were in the following order, respectively: Ext < Int-H1, Int-H2 < Int-O2 < Int-O1 and Int-O2 < Int-H2 < Ext < Int-H1, Int-O1 (α = 0.05). After 700 N vertical static loading, the removal torque values were statistically different from the initial values, and the post-loading values increased in the Int-O1 group and Int-H1 group (α = 0.05) and decreased in the Ext group, Int-H2 group, and Int-O2 group (α = 0.05). On the basis of the results of this study, it should be taken into consideration that a loss of the preload due to the settling effect can lead to screw loosening during a clinical procedure in the molar region where masticatory force is relatively greater.

## 1. Introduction

Attempts have been made to understand the factors that could compromise the settling effect of different implant abutment connections [1,2]. Various implant elements including the implant-abutment interface, the types of abutments, the screw characteristics, and the cyclic loading condition have all been shown to influence settling into implants and a loss of preload [3,4]. 

Loosening of the abutment screws and fixture failure in implant-supported restorations reportedly occur more frequently in the premolar and molar areas than in the incisor region [5,6]. This may result from differences in masticatory force and prosthetic design. Occlusion can be critical for implant longevity due to the nature of the potential load created by tooth contacts. The mechanism and vector of force transferred by posterior teeth differ from those of anterior teeth because posterior teeth have a stronger biting force in the vertical direction. Furthermore, these forces are produced by the action of the masticatory muscles [7].

The various forces that are exerted upon dental implants during function differ in magnitude and direction. In natural dentition, the periodontal ligament has the capacity to absorb stress and allow for tooth movement, but the bone-implant interface has little capacity to allow for the movement of an implant [8,9]. The force is distributed primarily along the crest of the ridge due to the lack of micromovement of implants [10].

Cyclic loading, which simulates functional loading, can significantly influence the overall intimacy of the settling of abutments into implants and their mechanical interlocking at the bone–implant interface [2]. However, cyclic loading is not the only factor that could influence the settling phenomenon in posterior teeth. Cyclic loading and the static loading are two independent conditions, and both can affect the settling of abutments into implants after occlusal loading.

In particular, vertical forces generated on implants in the posterior region are greatest at the implant-abutment interface. This means that vertical masticatory forces can affect settling into implants and a loss of preload after occlusal static loading.

Bruxism or clenching can create destructive lateral stresses and overloading when it transfers force to the supporting bone [11]. Parafunctional movements exert a greater maximum occlusal force than natural mastication. Van Eijden measured the mean magnitudes of a maximal vertical bite force in normal dentition without implants as follows: 469 ± 85 N at the canine region, 583 ± 99 N at the second premolar region, and 723 ± 138 N at the second molar region [12]. These results were comparable to the mean maximum bite force of 738 ± 209 N measured by Braun et al. [13]. In addition, Morneburg and Pröschel investigated vertical masticatory forces in vivo on implant-supported fixed partial dentures and found a mean total masticatory force of 220 N with a maximum of 450 N [14]. On the basis of these findings, the present study evaluated the degree of settling and compared preload loss using the removal torque values before and after 700 N static vertical loading.

The aim of this study was to evaluate the settling of abutments into implants and removal torque values of five different implant-abutment connections that differ significantly in macroscopic geometry after static vertical loading at 700 N.

## 2. Materials and Methods 

### 2.1. Implant-Abutment Systems Selection and Study Protocol 

**Figure 1:**
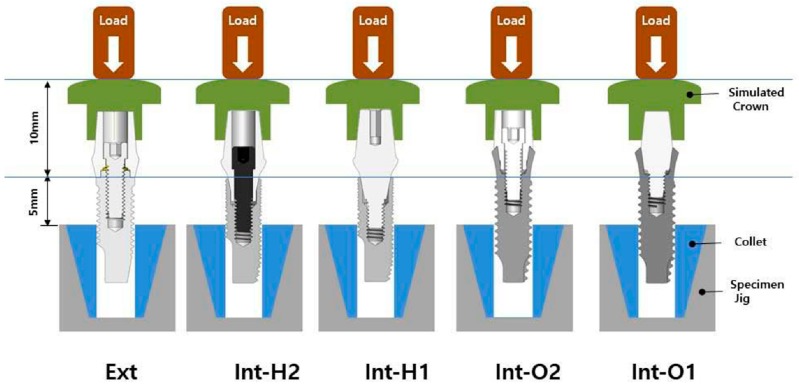
Schematic drawing of the test setup. Ext: external hexagon fixture + Cemented abutment; Int-H2: internal hexagon fixture + two-piece abutment; Int-H1: internal hexagon fixture + one-piece abutment; Int-O2: internal octagon fixture + two-piece abutment; Int-O1: internal octagon fixture + one-piece abutment.

One external and two internal connection implant systems from the Osstem Implant (Osstem Co., Seoul, Korea) were selected for the study. The abutment–implant assemblies were divided into five groups according to the implant connection designs and abutment types (Table 1, Figure 1).

Ext: External butt joint + Cemented abutment (two-piece)
Int-H2: Internal hexagon + Transfer abutment (two-piece)
Int-H1: Internal hexagon + Rigid abutment (one-piece)
Int-O2: Internal octagon + Comocta abutment (two-piece)
Int-O1: Internal octagon + Solid abutment (one-piece)

Ten implant-abutment assemblies were constructed for each group (total *n* = 50). Each assembly was held in a vise during the torque tightening procedure. The desired torque was applied to the abutment screw with a digital torque gauge (MGT12, MARK-10 Co., Hicksville, NY, USA).

**Figure 2:**
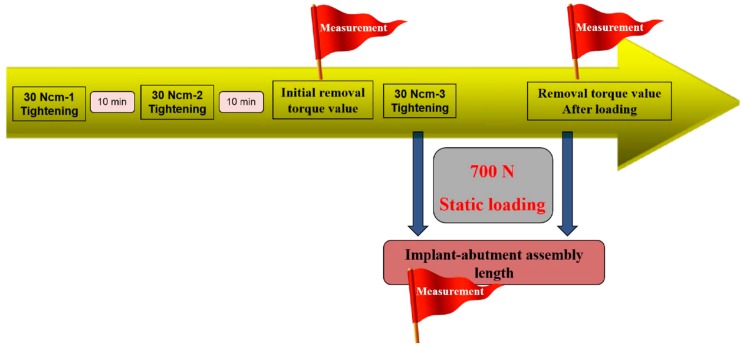
Schematic diagram of experimental design based on protocol sequence.

**Figure 3:**
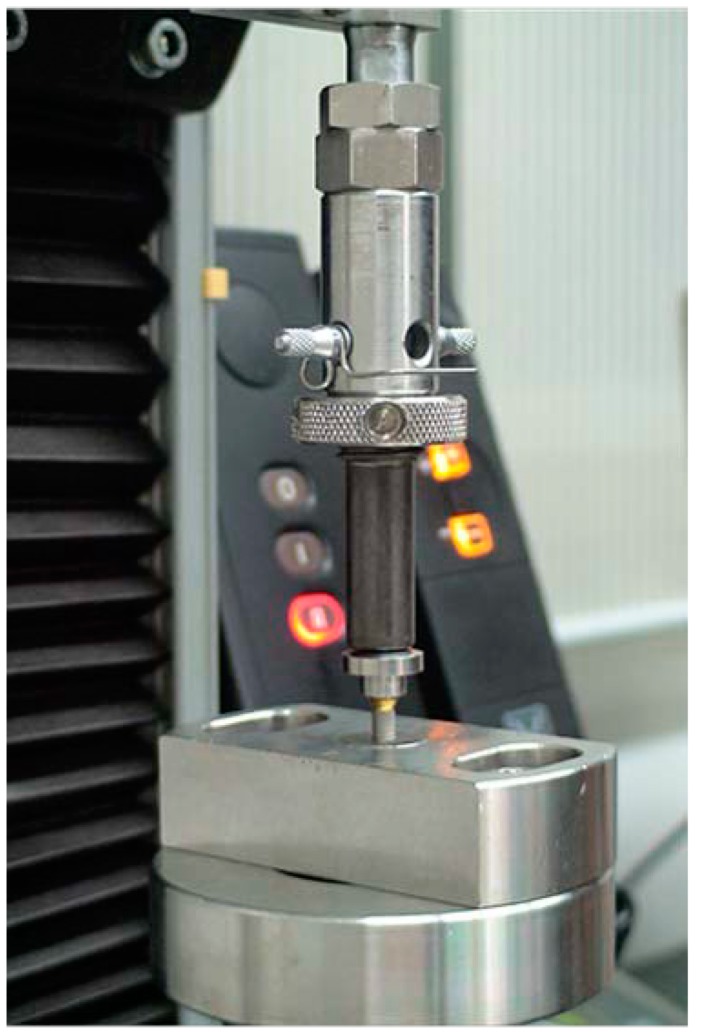
Loading machine and customized jig (Instron 8841, Instron Corp., Mass, Norwood MA, USA).

The schematic diagram of experimental design based on protocol sequence is presented in Figure 2. Each abutment was tightened into the corresponding implant at 30 Ncm torque twice at 10 minute intervals. Ten minutes after the second tightening, the initial removal torque was measured with a digital torque gauge (MGT12E, Mark-10 corp, Hicksville, NY, USA). Each assembly was secured again at 30 Ncm torque, and the total length of the implant-abutment assembly was measured with an electronic digital micrometer (no. 293-561-30, Mitutoyo, Japan). After the initial measurement of the total length, a metal cap fabricated to reproduce the crown was mounted on the abutment of the assembly and the entire unit was fixed in a loading jig (Figure 3). The loading jig was designed to withstand a 700 N vertical static force applied to the implant-abutment assembly. All the specimens were tested in a universal testing machine (Instron 8841, Instron Corp., Mass, Norwood MA, USA) under 700 N vertical static loading, corresponding to the maximum biting force in posterior teeth [12,13].

At the completion of static loading, the total length and removal torque of each implant-abutment specimen were measured in the same manner. The settling value of the abutment was calculated from the changes in the total lengths of the implant-abutment assembly before and after loading. The measurements were accurate up to 0.001 mm (1 μm) and the same operator performed all of the specimen preparations and testing in random order. The details of the experimental protocol and the overall outcomes between the magnitude of applied torque and the axil displacement of abutments into implants in external and internal implant-abutment connections were reported in previous studies [1,2].

### 2.2. Statistical Analysis

One-way ANOVA and Tukey’s honestly significant difference (HSD) tests were used to analyze settling lengths and removal torque of the five implant-abutment systems before and after 700 N vertical static loading. A paired *t*-test was performed to compare the initial and post-loading removal torques for each implant connection system. *p* < 0.05 was considered to represent a statistically significant difference. 

## 3. Results

**Figure 4:**
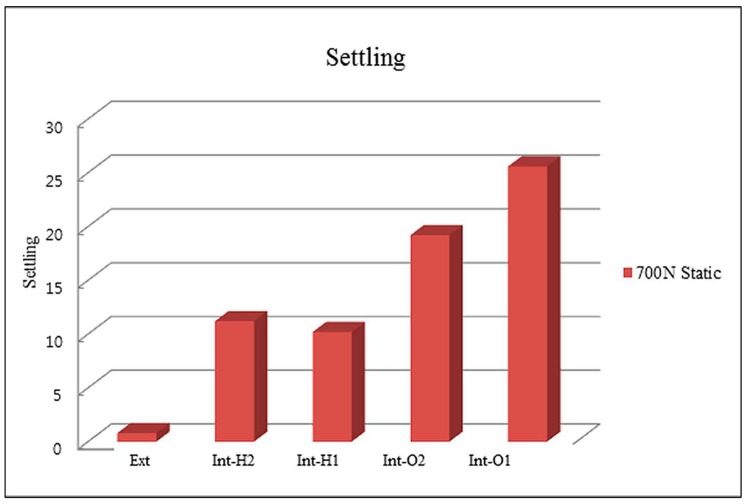
Settling of abutments into the implants after static loading (μm).

The mean lengths and settling values of the specimen groups after vertical static loading are presented in Table 2 and Table 3 and Figure 4. After 700 N static loading, there were statistically significant differences in the settling values in the Ext group (0.8 ± 0.45 μm), Int-H1 group (10.2 ± 0.84 μm), Int-H2 group (11.2 ± 0.84 μm), Int-O2 group (19.2 ± 4.21 μm), and Int-O1 group (25.6 ± 2.97 μm) (α = 0.05). In the internal octagon groups with an 8° Morse taper interface, there were greater increases compared with those seen in the other groups. A multiple comparison test by Tukey’s HSD exhibited differences in the settling values in each group after 700 N static loading in the following order: Ext < Int-H1, Int-H2 < Int-O2 < Int-O1 (see Table 2 and Table 3). 

**Figure 5:**
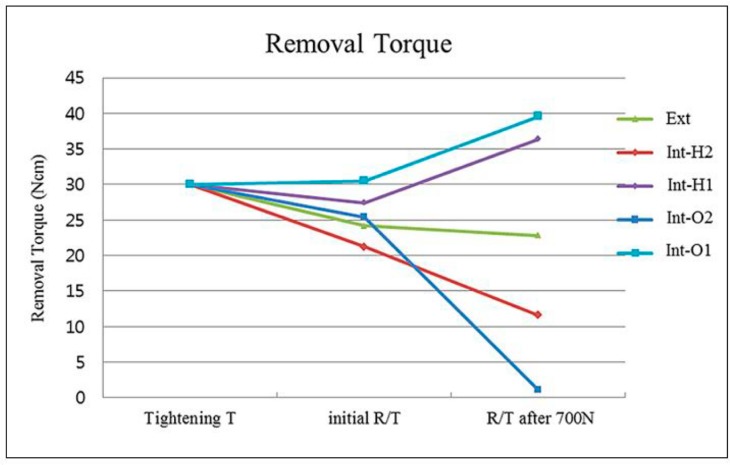
Removal torque (Ncm) after 700 N static loading.

The mean values of removal torque after loading are presented in Table 4, Table 5 and Table 6 and Figure 5. After 700 N static loading, the Int-O1 group exhibited the highest removal torque of 39.64 ± 4.28 Ncm. The other groups are shown in the following decreasing order: Int-H1 (36.38 ± 6.25 Ncm), Ext (22.78 ± 0.40 Ncm), Int-H2 (11.62 ± 0.56 Ncm), and Int-O2 (1.14 ± 0.40 Ncm). Using Tukey’s HSD, the specific group-wise comparisons in the post-loading removal torque values were as follows: Int-O2 < Int-H2 < Ext < Int-H1, Int-O1. 

In cases in which one-piece abutments were used for the internal connection system (Int-H1 group and Int-O1 group), the removal torque was increased compared to the initial removal torque. In cases where two-piece abutments were used for the internal connection system (Int-H2 group and Int-O2 group), after 700 N vertical static loading, the removal torque was decreased compared to the initial removal torque to a greater extent. In the Int-O2 group in particular, the abutment screw nearly came loose from the abutment. After 700 N loading, the removal torque value also exhibited a small but significant decrease in the Ext group (Table 6).

## 4. Discussion

Along with the expanded indications for implants and the changing clinical protocols, the relationship between implant design and load distribution at the implant–bone interface has become an important issue. The inadequate interaction between these two factors may result in both mechanical and biologic complications such as screw loosening and peri-implant bone loss. Whether an implant prosthesis is placed in function after an undisturbed healing period or immediately after placement, the biomechanical environment is, thereafter, a critical factor that influences implant duration and bone preservation. Loads applied to teeth and implants during physiologic oral functions including chewing, clenching, swallowing, or grinding may vary because the anchorage of natural and artificial abutments in the jaw is not of the same type and quality [15].

Most of the studies related to axial displacement [1,2,3] are on the magnitude of tightening torque and the duration of cyclic loading, and few studies have applied with static loading. Ko et al. [4] reported that axial displacement and reverse torque loss occurred at significantly low levels after the cyclic and static loading in the case of wide-type implants of 5.0 mm diameter. In addition, the CAD/CAM (Computer Aided Design/Computer Aided Manufacturing) customized abutments, which are currently in the spotlight, may show differences in the fabricating process from the stock abutments produced by manufacturers. Therefore, using implant fixtures and abutments made by the same manufacturer, we wanted to prove that axial displacement could occur even at static loading of 700 N, and the difference comes from different connection types.

For osseointegrated dental implants, previous studies have revealed that occlusal interferences and parafunctional activities may lead to mechanical and biologic complications [16]. Many investigators have attempted to evaluate maximum bite forces. Typical maximum bite force magnitudes exhibited by adults are affected by age, sex, degree of edentulism, bite location, and especially parafunction. In centric occlusion involving swallowing and clenching, forces are transmitted bilaterally, predominantly by molars and premolars. For a single tooth or implant in the molar region, the greatest forces occur along the axial direction [17]. Therefore, the results of this study showed the settling effect in relation to a loss of removal torque after 700 N vertical static loading, corresponding to the maximum masticatory force. 

The settling effect after 700 N loading showed a clinical association between screw loosening with a loss of preload and an increase in friction. The results followed a similar pattern with cyclic loading in our previous study [2]. The Ext group showed the lowest settling due to its flat platform interface. Likewise, the internal hexagon and octagon groups had statistically greater settling due to their tapered interface. In particular, the internal octagon group with an 8° Morse taper showed the highest settling value compared to the internal hexagon group with an 11° taper. 

The removal torque values after 700 N vertical static loading may be influenced by the amount of settling and the type and configuration characteristics of the abutment used. When a two-piece abutment, as seen in the Int-H2 and Int-O2 groups, is used, the screw joint connection is based on the tension mechanism, where a screw may become loose due to a loss of preload by settling. Therefore, the settling effect of the Int-H2 and Int-O2 groups produced a significant decrease in the removal torque even to the extent of the loss of the abutment screw in the Int-O2 group. On the other hand, when a one-piece abutment is used, the main retention mechanism is friction. As a result, the settling effect of the one-piece abutment in the Int-H1 and Int-O1 groups created a greater compressive force at the implant-abutment interface, which resulted in the increased post-loading values of removal torque. 

The metal cap used in this experimental protocol was inserted into the abutment by friction only, and without dental cement. The simulated crown had a gap between the abutment and the metal cap in order to prevent any forces from being transferred to the abutment during the removal of the crown. However, because the margin of the crown was seated on the fixture in the original internal octagon design, there was no such space. Consequently, this discrepancy may have led to greater settling values than the actual value due to the lack of a vertical stop. In addition, this study could not use the direct method as described by Haack et al., where the change in the preload was evaluated by measuring the length of an elongated screw [18]. Therefore, further studies are warranted to evaluate the actual measurement of an elongated screw as a value of tightening torque. 

## 5. Conclusions

The current study strived to gain a better understanding of the nature of the implant-abutment screw joint on the basis of the settling effect and removal torque. On the basis of the findings of this study, in the molar region where masticatory force is relatively greater, a loss of preload due to the axial displacement and the possibility of screw loosening should be taken into account in clinical procedures. 

The clinical implication of this study is that when the implant fixture of a regular platform with a diameter of 4.0 mm is placed in the posterior molar region, the settling of abutments into implants caused by the vertical force may cause a problem of lowering the occlusion after the prosthesis is mounted.

## Figures and Tables

**Table 1:** Characteristics of experimental implant-abutment systems.

Group	Ext	Int-H2	Int-H1	Int-O2	Int-O1
Implant system	US II	GS II		SS II	
Implant/abutment interface	External butt joint	11° taper internal hexagon		8° morse taper internal octagon	
Abutment type	Cemented (two-piece)	Transfer (two-piece)	Rigid (one-piece)	Comocta (two-piece)	Solid (one-piece)
Abutment material	Ti CP-Gr 3	Ti CP-Gr 3	Ti-6Al-4V	Ti CP-Gr 3	Ti-6Al-4V
Abutment diameter	Ø5.0	Ø5.0	Ø5.0	Ø4.3	Ø3.5
Abutment gingival height	2 mm	2 mm	2 mm	-	-
Abutment height (H_A_)	5.5 mm	5.5 mm	5.5 mm	4 mm	4 mm
Abutment screw	Ta	WC/C Ta	-	Ta	-
Fixture material	Ti CP-Gr 4	Ti CP-Gr 4		Ti CP-Gr 4	
Fixture diameter	Ø4.0	Ø4.0	Ø4.0	Ø4.1	Ø4.1
Fixture height(H_F_)	11.4 mm	11.5 mm	11.5 mm	11.5 mm	11.5 mm
Feature	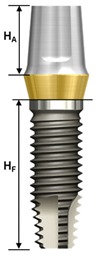	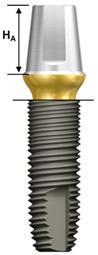	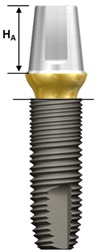	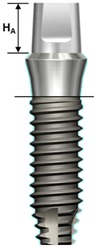	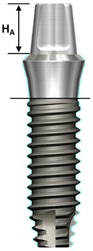

Ext: external hexagon fixture + Cemented abutment; Int-H2: internal hexagon fixture + two-piece abutment; Int-H1: internal hexagon fixture + one-piece abutment; Int-O2: internal octagon fixture + two-piece abutment; Int-O1: internal octagon fixture + one-piece abutment; Ta: titanium alloy; WC/C Ta: tungsten carbide/carbon-coated titanium alloy; H_A_: Abutment height; H_F_: fixture height.

**Table 2:** Mean total lengths and standard deviations of the implant-abutment specimens before and after 700 N static loading.

Group	Ext (mm)	Int-H2 (mm)	Int-H1 (mm)	Int-O2 (mm)	Int-O1 (mm)
Tightening torque	18.6096	18.9624	19.0456	18.9564	18.9992
30 Ncm-③ *	±0.0054	±0.0153	±0.0261	±0.0222	±0.0041
Load 700 N Static **	18.6088	18.9512	19.0354	18.9372	18.9736
	±0.0054	±0.0151	±0.0266	±0.0222	±0.0035

* Additional tightening at 30 Ncm after measuring the initial removal torque after the second 30 Ncm tightening. ** After 700 N vertical static loading.

**Table 3:** Mean settling values after 700 N static loading in each group and multiple comparisons using Tukey’s honestly significant difference (HSD).

Group	Settling Values Mean ± SD (μm)	Group Comparisons †
Ext	0.8 ± 0.45	Ext < Int-H1, Int-H2 < Int-O2 < Int-O1 Settling value = (total lengths of the implant-abutment assemblies at 30 Ncm-③) minus (total lengths of the implant-abutment assemblies after 700 N static loading)
Int-H2	11.2 ± 0.84	
Int-H1	10.2 ± 0.84	
Int-O2	19.2 ± 4.21	
Int-O1	25.6 ± 2.97	

† Tukey’s HSD method was performed for between group comparisons (*p* < 0.05).

**Table 4:** Multiple comparisons of mean values of initial removal torque and removal torque after 700 N static loading.

Test	Group (*n* = 5)	Tightening Torque (Ncm)	Removal Torque (Ncm)	Significance †
Initial removal torque	Ext	30	24.22 ± 0.81	Int-H2 < Ext, Int-O2 < Int-H1 < Int-O1
	Int-H2	30	21.22 ± 1.04	
	Int-H1	30	27.44 ± 0.92	
	Int-O2	30	25.38 ± 1.86	
	Int-O1	30	30.54 ± 0.56	
Removal torque after 700N static loading	Ext	30	22.78 ± 0.40	Int-O2 < Int-H2 < Ext < Int-H1, Int-O1
	Int-H2	30	11.62 ± 0.56	
	Int-H1	30	36.38 ± 6.25	
	Int-O2	30	1.14 ± 0.40	
	Int-O1	30	39.64 ± 4.28	

† Tukey’s HSD method was performed for between group comparisons (*p* < 0.05).

**Table 5:** Comparison of the mean values of initial and post-loading removal torque in each group.

Group	Initial R/T ^a^	R/T after Static Load ^b^	Significance †
Ext	24.22 ± 0.81	22.78 ± 0.40	*
Int-H2	21.22 ± 1.04	11.62 ± 0.56	**
Int-H1	27.44 ± 0.92	36.38 ± 6.25	NS
Int-O2	25.38 ± 1.86	1.14 ± 0.40	*
Int-O1	30.54 ± 0.56	39.64 ± 4.28	*

^a^ Removal torque values before loading^; b^ Removal torque values 700 N static loading; † Paired *t*-test was performed to compare the removal torque values before and after loading: NS, not significant; * *p* < 0.01; ** *p* < 0.001.

**Table 6:** Comparisons of the mean values of initial removal torque and removal torque after static loading in each group.

Group	Removal Torque	*t/P*
		Value
Ext	Initial	6.279
	after 700 N static loading	/0.003 *
Int-H2	Initial	16.204
	after 700 N static loading	/0.000 *
Int-H1	Initial	−3.313
	after 700 N static loading	/0.030 *
Int-O2	Initial	6.413
	after 700 N static loading	/0.003 *
Int-O1olid	Initial	−4.768
	after 700 N static loading	/0.009 *

* indicates values that were statistically different (*p* < 0.05).
